# English Doctors Abroad

**Published:** 1897-07-03

**Authors:** 


### The Hospital

A Journal of

The Medical Sciences and Hospital Administration.

Vol. XXII.—No. 562.] [July 3, 1897.

### English Doctors Abroad.

During the recently terminated session of the General Medical Council, a letter was read from Mr. Eyre, a medical practitioner, deriving his qualifications from Dublin and Cambridge, and settled in Rome, on a project of law which has been presented to the Italian Chamber by the Government, and which, therefore, is very likely to be adopted. By this project, as described in Mr. Eyre's letter, which does not give its precise terms, it would appear that foreign medical men, that is to say, persons who are not of Italian nationality and who do not possess Italian medical qualifications, will be deprived of the privilege which they now possess of professionally attending other foreigners who may be either resident in or passing through Italy. It will be within the knowledge of our readers that laws prohibiting the medical practice of foreigners with foreign qualifications exist in almost all European countries, and have only recently been passed in some of them; and it would seem that Italy desires to bring herself into line with the rest in this respect. The General Medical Council, having considered Mr. Eyre's letter, resolved to appoint a committee to prepare and forward a representation on the subject to the Privy Council, with a request that the Secretary of State for Foreign Affairs might be moved to take such steps for the protection of British practitioners abroad as he might think practicable or desirable. No reply to this representation can be received, or, at least, none can be made public, until the next session of the Council in November; but it may safely be assumed that the committee has already taken the action indicated by the resolution of the Council, and that the attention of the Government will at least be called to the merits and to the urgency of the case.

It is not too much to affirm that nearly all the so-called "health resorts" in Italy, France, and Switzerland are indebted for the public appreciation, if not for the actual discovery, of their several advantages to the energy, and often to the capital, of English medical settlers, with the natural result that English invalids have flocked to these places season after season in considerable numbers, and have spent much money in them, in no small degree on account of their having been able until recently to obtain the advice of English medical practitioners, conversant with the habits of life of English people and with the methods of treatment suited to them. Moreover, the English practitioners in question were, as a rule, well known to a large number of their professional brethren in this country, from whom they were accustomed to receive, together with patients, valuable information about the maladies from which these patients were suffering, and about any personal or family peculiarities which required to be remembered with regard to them. In this way the change of air and scene, or the other advantages of the Continental health resort, were obtained without any break in the continuity of the treatment, and the returning patient brought back, not only improved health, but also full information to his or her accustomed physician. It was not unimportant that the possession of a common language afforded—both to patient and doctor—a protection against mistakes and misconceptions, which might entail disaster upon the hapless invalids who might suffer from their effects.

In many parts of Europe all this has been changed, and it seems likely to be changed also in Italy. The truth is that native doctors in France and Switzerland have for a long time looked with greedy eyes at the incomes supposed to be earned in those countries by Englishmen for medical attendance upon their own countrymen and countrywomen, and that they have now played a bold stroke for the purpose of securing these incomes for themselves. They say, in effect, to the English invalid, "you shall not have our baths, or our springs, or our climates, unless you also have *us*." In this country, doctors have managed their political affairs badly, and have failed to secure the influence which they have succeeded in securing abroad. The result is that foreign politicians are mindful of the prosperity of foreign doctors, while English politicians are comparatively indifferent to the prosperity of English ones. At the present time, if her Majesty were to return to Cimiez with an English doctor in her suite, that doctor would incur legal penalties if he were to prescribe for his illustrious patient. That such penalties would probably not be enforced does not alter the question; and it is a fact that an English doctor travelling in Switzerland has lately been threatened with punishment for attending his own mother in the hotel at which he was staying with her. The case seems to be one, if ever there was one, in which reprisals would be legitimate; and the home Government, if it attached proper importance to the protection of English subjects, would pass an Act to forbid medical practice in Great Britain by foreigners holding only foreign qualifications, but reserving power to the Privy Council to except the natives of any colonies or countries which, within their own boundaries, granted reciprocal privileges to Englishmen. Our corporations should at the same time abandon their present custom of rendering the examination of a duly qualified foreigner for an English diploma a matter almost of form, and should insist upon an entire curriculum and upon a rigid standard of acquirement. It is said that, in the countries from which English practitioners have recently been excluded, care would now be taken to reject any one of them who might endeavour to maintain his position by passing the examination for any native diploma.

